# Evaluation of the safety and efficacy of fecal microbiota transplantations in bottlenose dolphins (*Tursiops truncatus*) using metagenomic sequencing

**DOI:** 10.1093/jambio/lxae026

**Published:** 2024-02-01

**Authors:** Barbara K Linnehan, Sho M Kodera, Sarah M Allard, Erin C Brodie, Celeste Allaband, Rob Knight, Holly L Lutz, Maureen C Carroll, Jennifer M Meegan, Eric D Jensen, Jack A Gilbert

**Affiliations:** National Marine Mammal Foundation, San Diego, CA 92106, United States; Scripps Institution of Oceanography, University of California San Diego, La Jolla, CA 92037, United States; Scripps Institution of Oceanography, University of California San Diego, La Jolla, CA 92037, United States; Department of Pediatrics, University of California San Diego School of Medicine, La Jolla, CA 92093, United States; National Marine Mammal Foundation, San Diego, CA 92106, United States; Department of Pediatrics, University of California San Diego School of Medicine, La Jolla, CA 92093, United States; Department of Pediatrics, University of California San Diego School of Medicine, La Jolla, CA 92093, United States; Center for Microbiome Innovation, Joan and Irwin Jacobs School of Engineering, University of California San Diego, La Jolla, CA 92093, United States; Department of Medicine, University of California San Diego, La Jolla, CA 92161, United States; Department of Computer Science and Engineering, University of California San Diego, La Jolla, CA 92093, United States; Department of Bioengineering, University of California San Diego, La Jolla, CA 92093, United States; Department of Immunology and Microbiology, Scripps Research Institute, La Jolla, CA 92037, United States; MSPCA Angell Animal Medical Center, Boston, MA 02130, United States; National Marine Mammal Foundation, San Diego, CA 92106, United States; U.S. Navy Marine Mammal Program, Naval Information Warfare Center Pacific, San Diego, CA 92106, United States; Scripps Institution of Oceanography, University of California San Diego, La Jolla, CA 92037, United States; Department of Pediatrics, University of California San Diego School of Medicine, La Jolla, CA 92093, United States; Center for Microbiome Innovation, Joan and Irwin Jacobs School of Engineering, University of California San Diego, La Jolla, CA 92093, United States

**Keywords:** *Tursiops truncatus*, microbiome, fecal microbiota transplantation, shotgun metagenomics

## Abstract

**Aims:**

Gastrointestinal disease is a leading cause of morbidity in bottlenose dolphins (*Tursiops truncatus*) under managed care. Fecal microbiota transplantation (FMT) holds promise as a therapeutic tool to restore gut microbiota without antibiotic use. This prospective clinical study aimed to develop a screening protocol for FMT donors to ensure safety, determine an effective FMT administration protocol for managed dolphins, and evaluate the efficacy of FMTs in four recipient dolphins.

**Methods and Results:**

Comprehensive health monitoring was performed on donor and recipient dolphins. Fecal samples were collected before, during, and after FMT therapy. Screening of donor and recipient fecal samples was accomplished by in-house and reference lab diagnostic tests. Shotgun metagenomics was used for sequencing. Following FMT treatment, all four recipient communities experienced engraftment of novel microbial species from donor communities. Engraftment coincided with resolution of clinical signs and a sustained increase in alpha diversity.

**Conclusion:**

The donor screening protocol proved to be safe in this study and no adverse effects were observed in four recipient dolphins. Treatment coincided with improvement in clinical signs.

Impact Statement Fecal microbiota transplantation holds promise in dolphin medicine as a therapeutic tool to restore healthy gut microbiota without antibiotic use.

## Introduction

Gastrointestinal illnesses are one of the top causes of morbidity in bottlenose dolphins (*Tursiops truncatus*) under managed care. Such conditions are often associated with changes in the gut microbiome that indicate disruption of the symbiotic equilibrium between the microbiome and the host. About 80% of the mammalian immune system resides in the intestines and changes in its microbiome can negatively affect host health through inflammatory disorders (Chassaing et al. 2014, Wiertsema et al. 2021). Effects can reach far beyond the gastroinestinal (GI) tract to include neurodegenerative disease, metabolic disease, depression, cardiopulmonary disease, autoimmune disorders, and others (Carabotti et al. 2015, Tremlett et al. 2017). Although the factors that lead to microbial community disruption are still under investigation, changes in diet, use of pharmacological agents, stress, and disease status have all been shown to impact the microbiome and may lead to problems in host health (Lozupone et al. 2012, Imhann et al. 2016, Lloyd-Price et al. 2016, Suchodolski 2016, Kostrzewska et al. 2017, Koo et al. 2019, Suchodolski 2022).

Recently, fecal microbiota transplants (FMTs) have shown promising results as a potential therapeutic tool. In human medicine, there are a myriad of studies demonstrating the effectiveness of FMTs in treating various diseases, with beneficial effects ranging from improved immune function to improved cognitive and mental health (Choi and Cho 2016, Kang et al. 2019, Hamamah et al. 2022). FMT is most commonly utilized in humans for the treatment of recurrent *Clostridium difficile* infections. The U.S. Food and Drug Administration recently approved a rectal fecal microbiota product (REBYOTA^®^, Ferring Pharmaceuticals Inc.) in 2022 and an oral fecal microbiota capsule product (VOSWT, Seres Therapeutics Inc.) in 2023, both to treat recurrent *C. difficile* infection. In veterinary medicine, FMT science is still in its infancy and is not yet widely used, perhaps due to safety concerns and lack of understanding of its therapeutic mechanisms (Tuniyazi et al. 2022). Yet, there are a growing number of canine, large animal, and laboratory animal studies demonstrating the benefits of FMT therapy to treat a wide array of gastrointestinal diseases (Murphy et al. 2014, Minamoto et al. 2015, Burton et al. 2016, Hensley-McBain et al. 2016, Furmanski and Mor 2017, Smith et al. 2017, Yamazaki et al. 2017, Dias et al. 2018, Niederwerder 2018, Pereira et al. 2018, Greene et al. 2019, Niina et al. 2019, 2021, Sugita et al. 2019, McKinney et al. 2020, Costa et al. 2021, Diniz et al. 2021, Gal et al. 2021, Kim et al. 2021, Laustsen et al. 2021, Brown et al. 2022, Tuniyazi et al. 2022, Tuniyazi et al. 2023). For example, applications of FMT treatment in canines have been shown to treat refractory *C. perfringens* diarrhea (Murphy et al. 2014), acute hemorrhagic diarrhea syndrome (Gal et al. 2021), inflammatory bowel disease (Minamoto et al. 2015, Niina et al. 2019, 2021), *C. difficile* infection (Sugita et al. 2019, Diniz et al. 2021), parvovirus (Pereira et al. 2018), and postweaning diarrhea (Burton et al. 2016). There are two peer-reviewed studies in aquatic species, including a report of oral FMT treatment in a pilot whale with recurrent gastrointestinal disease, which demonstrated initial resolution of dysbiosis and associated clinical signs but subsequent relapse and illness (Brown et al. 2022). A study in African killifish demonstrated improved lifespan and delayed behavioral decline when young donor feces were transplanted to older fish (Smith et al. 2017).

Data regarding the use of FMT in dolphins to treat dysbiosis or chronic GI disease have previously been limited to a handful of anecdotal cases with no standardized dosing, frequency, or metrics to measure efficacy. Knowledge of the normal gastrointestinal microbiota in dolphins is limited and clinical tools to monitor GI disease or dysbiosis even more so. The dolphin microbiome has been shown to be distinct from the surrounding aquatic environment (Bik et al. 2016, Robles-Malagamba et al. 2020) and has been described in managed and wild dolphins (Bik et al. 2016, Soverini et al. 2016, Suzuki et al. 2019, Robles-Malagamba et al. 2020); it has been shown to differ from that of terrestrial carnivores and more closely resembles that of marine piscivores (Bik et al. 2016, Soverini et al. 2016). Differences in diet and environment may also largely contribute to differences in the dolphin gut microbiome across locations (Suzuki et al. 2019), confounding efforts to characterize healthy dolphin microbiome states. Moreover, dolphins do not have firm feces like domestic mammals, causing challenges with observing abnormal defecation in dolphins and with donor fecal collection for FMT treatment.

In both human and animal studies, there is not a single established protocol for FMT administration, and many studies are contradictory. Existing protocol variations include applications of a single FMT versus a series of repeated FMTs (Quraishi et al. 2017, Allegretti et al. 2018, Ianiro et al. 2018, Sugita et al. 2019, Chaitman et al. 2020, Roshan et al. 2020, Gal et al. 2021, Zou et al. 2022); utilization of rectal/endoscopic routes of delivery versus oral routes (Kassam et al. 2013, Minamoto et al. 2015, Krajicek et al. 2019, Sugita et al. 2019, Diniz et al. 2021); and the use of fresh donor feces versus frozen feces (Satokari et al. 2015, Quraishi et al. 2017, Staley et al. 2017, Ianiro et al. 2018, Krajicek et al. 2019). Standardized protocols for safe FMT use in dolphins are necessary, as well as more sensitive tools for screening donors and monitoring outcomes. To address these needs, the goals of this prospective clinical study were to (i) develop a screening protocol for FMT donor dolphins to ensure safety, (ii) develop an effective FMT administration protocol for use in managed bottlenose dolphins, and (iii) examine the efficacy of FMT therapy in four dolphins with enteropathies utilizing shotgun metagenomic sequencing. As the majority of intestinal bacteria cannot be cultured by traditional methods, molecular tools such as shotgun metagenomic sequencing allow for a comprehensive analysis of intestinal microbiota and can expand our understanding of the taxa present far beyond that reported from culture-dependent methods alone.

## Materials and methods

### Animals

Fourteen bottlenose dolphins living in open ocean enclosures and cared for by the US Navy Marine Mammal Program (MMP) in San Diego, California, participated in the prospective clinical study from January 2019 to February 2022 (age range 6–47 years, 13 males and 1 female). The dolphins are trained for husbandry behaviors using operant conditioning and participated voluntarily in all steps of their care. Samples from animals were collected during their routine clinical care and under the authority codified in the US Code, Title 10, Section 7524. Secretary of Navy Instruction 3900.41H directs that Navy marine mammals be provided the highest quality of care. The MMP is accredited by AAALAC International and adheres to the national standards of the US Public Health Service Policy on the Humane Care and Use of Laboratory Animals and the Animal Welfare Act. The dolphins are fed mixtures of quality-controlled, frozen-thawed fish and additional vitamin supplements (Vita-Zu Mammal Tablet # 5M26, Mazuri, Richmond, IN, USA). Fish types fed during the study period, included capelin (*Mallotus villosus*), Pacific herring (*Clupea pallasii*), Pacific squid (*Loligo opalescens*), ballyhoo (*Hemiramphus brasiliensis*), pinfish (*Lagodon rhomboides*), and Atlantic threadfin herring (*Opisthonema oglinum*).

### Donors

Seven dolphins were included in the study as healthy donors, including six males and one female (ages 7, 9, 16, 16, 20, 39, and 46 years). Inclusion criteria for FMT donor dolphins were defined as dolphins who were deemed healthy based on routine physical exam, full body ultrasound, and blood analysis (complete blood count, serum chemistry, erythrocyte sedimentation rate, and fibrinogen), with no known illness within the last 6 months, and not receiving any antibiotics, antifungals, or probiotics within the last 6 months or at any point during the study. In addition, donor fecal samples were thoroughly analyzed following the protocol below to ensure safety. Dolphins with abnormal results on the screening tests were excluded as donors.

### FMT recipient dolphins

Recipient dolphins were selected based on having a history of diarrhea, poor appetite, or abnormal behavior during the baseline analysis, and all received antibiotics at a young age. Due to delays between the baseline analysis and FMT therapy, at the time of the FMT trials, only one dolphin had active clinical signs in this study (Dolphin A). The other three recipients were still administered the FMT therapy despite lack of active clinical signs due to cytological evidence of bacterial imbalance and history of early antibiotic use. Previous studies have shown benefits of restoring healthy flora in young animals with a history of antibiotic use (Lynn et al. 2021, Ma et al. 2022).

#### Recipient Dolphin E (pilot case: clinical at time of first FMT; subclinical at time of this study)

Dolphin E, a 4-year-old male bottlenose dolphin, developed gastroenteritis in June–July 2019, which was diagnosed as an enteric coronavirus on the University of Georgia Veterinary Diagnostic Laboratories PCR diarrhea panel (canine puppy diarrhea panel). The virus was later sequenced from feces as a novel bottlenose dolphin coronavirus (BdCoV) (Wang et al. 2020). After the initial acute diarrhea and inappetence, the animal developed chronic diarrhea, with waxing and waning inappetence, lethargy, and lack of participation in voluntary behaviors. He was treated with supportive care (antinausea, antiemetics, and fluid support) and with antimicrobials when he developed leukocytosis and an inflammatory hemogram. Due to the chronic diarrhea, lethargy, and inappetence, FMTs were initiated in August 2019; he received FMTs once every week for 4 weeks, then every-other-week for four treatments, then monthly until December 2019. During the course, his diarrhea, anorexia, and lethargy resolved, and he was successfully weaned off of all medications. As this FMT treatment was performed prior to the conception of the FMT study, fecal samples from this first round of FMTs were not banked for metagenomic analysis and, therefore, were not included in this study. Given the clinical success achieved in this pilot dolphin with FMTs, this prospective clinical study to better evaluate the donor screening protocol and outcomes was undertaken.

Following the pilot FMT course in 2019, Dolphin E also participated as a recipient during this study in 2020–2021. At the start of the study, he was not on any medications, had a normal appetite, normal energy levels, and was not experiencing any diarrhea.

#### Recipient Dolphin C (subclinical at the time of FMT)

Dolphin C, a 13-year-old male bottlenose dolphin, was first treated with antibiotics at 9-year-old for pneumonia. Following this treatment, he developed enteritis, characterized by monomorphic cocci on cytology, and intermittent diarrhea. At this time, he also had difficulty with some task-based learning and was treated with oral diazepam to facilitate appetite and focus, with varying success. He had recurrent *Clostridial* overgrowths on culture, with positive *C. perfringens* enterotoxin PCR [University of Georgia Veterinary Diagnostic Laboratory PCR diarrhea panel (canine puppy diarrhea panel)]. By the time of the FMT therapies in this study, Dolphin C was no longer on any medications, had a normal appetite, normal stool consistency, and trainers saw improvements in task-based learning.

#### Recipient Dolphin Z (subclinical at the time of FMT)

Dolphin Z, a 6-year-old male bottlenose dolphin, had a history of antibiotic use as a calf due to inflammatory hemograms and skin wounds. At 3-year-old, he required intravenous antibiotics to treat pneumonia. Following this treatment, he developed enteritis, characterized by monomorphic cocci on cytology and evidenced by chronic, intermittent diarrhea. He also had recurrent *Clostridial* overgrowths, with positive *C. perfringens* enterotoxin PCR. By the time of the FMT therapies in this study, Dolphin Z was stable with a normal appetite and stool consistency. He also showed improvement in task-based learning, and he was no longer on any medications.

#### Recipient Dolphin A (clinical signs at the time of FMT)

Dolphin A, a 6-year-old male bottlenose dolphin, was not initially part of the study; however, after the baseline and FMT phases of this study were completed, Dolphin A developed gastroenteritis in August 2021. The animal was diagnosed with adenovirus via positive PCR of feces. He subsequently developed leukopenia and required intravenous antibiotics and supportive care. He recovered from the acute incident but developed chronic diarrhea afterwards. When supportive medications were tapered, appetite would acutely decrease. Fecal cytology showed a monomorphic population of cocci. FMT therapy began in November 2021 and continued through February 2022. He received FMTs from the same donors as the other three recipients in the study, but given the different timeframe of his FMTs, the exact composition of the donor slurries differed.

#### Control dolphins

Three control dolphins were included in the study; fecal samples were collected along the same timeline and in the same manner as the recipient dolphins, but no FMTs were performed. Two of these dolphins were healthy controls (Dolphin L and Dolphin I, adult males, ages 19 and 23 years). One of the dolphins had a history of a single, acute, self-limiting episode of bacterial enteritis but never received FMTs (Dolphin N, 18-year-old male). This dolphin had an episode of diarrhea in November 2020 (6 months before sample collection began); fecal culture grew heavy *C. perfringens*, and was positive for *C. perfringens* Toxin A on PCR. He did not receive antibiotic therapy.

### Fecal collection

Fecal samples were collected from dolphins in routine fashion: under voluntary behavioral control, the dolphins assumed a ventral presentation in the water with a trainer supporting the peduncle. A sterile, 25-inch, 14Fr catheter (suction catheter with the suction adapter removed; JorVet, Jorgensen Labs, CO, USA) was advanced into the rectum using sterile lubricant (Surgilube^®^, HR Pharmaceuticals, Inc., York, PA, USA), ∼20 inches deep. A syringe was attached to the end of the catheter and gentle suction applied for fecal collection, with caution to not apply too much suction and damage the colonic mucosa.

### Donor fecal screening protocol

Fecal samples from prospective donor dolphins were thoroughly screened to ensure safety and minimize the risk of transplantation of enteropathogenic microbes or viruses. Every 3–6 months, one fecal sample from each potential donor dolphin was analyzed with in-house cytology, in-house fecal float to examine for ova/parasites (Fecasol^®^ sodium nitrate solution, Vetoquinol USA, Inc.), aerobic, anaerobic, and fungal cultures (University of Illinois Veterinary Diagnostic Lab), and a PCR diarrhea panel, including *Campylobacter jejuni/coli, Salmonella* spp., *Lawsonia intracellularis, C. difficile* enterotoxin A, *C. difficile* cytotoxin B, *C. perfringens* enterotoxin, *Giardia intestinalis*, coronavirus, and adenovirus (Canine Puppy Diarrhea Panel plus adenovirus; University of Georgia Veterinary Diagnostic Lab, Athens, GA, USA). Dolphins with any positive results on the PCR diarrheal panel or positive results for *Clostridial* or *Escherichia coli* enterotoxins from culture were excluded as donors in the initial screening prior to beginning this study. Eleven dolphins were screened as donors; the seven donor dolphins in this study passed this screening protocol, while four others were excluded due to subsequent antimicrobial use (*n = *3) and positive *C. perfringens* enterotoxin PCR (*n = *1). In-house cytology was performed weekly on donor samples during the FMT process to monitor for adverse changes.

Additionally, metagenomic sequencing was utilized as an additional screen of potential donor feces for significant relative abundances of known or suspected bacterial pathogens. The compiled list of known or suspected bacterial pathogens based on available bottlenose dolphin literature included *Erysipelothrix rhusopathiae, Brucella ceti, Mycobacterium marinum, M. chelonae, M. abscessus, Nocardia asteroides, N. farcinica, N. brasiliensis, N. cyriacigeorgica, and N. levis, Salmonella* spp., *Staphylococcus aureus, Streptococcus phocae, Strep. zooepidemicus, Strep. iniae, Mycoplasma* spp., *Bartonella* spp., *Clostridium* spp., and *E. coli* (Venn-Watson et al. 2008, 2012, Morris et al. 2011, Bik et al. 2016, Soverini et al. 2016, Terio et al. 2018).

When extra feces (≥5 ml) were collected from donors beyond that needed for screening tests, it was banked for future potential donation. The fecal samples were combined with 5 ml of 10% glycerol solution (Fisher Scientific, Waltham, MA, USA) and banked at −80ºC, following standard protocol to improve survival of microbes (Satokari et al. 2015).

### Phase 1: baseline analysis (pre-FMT)

From January to July 2020, three baseline fecal samples from each donor and recipient dolphin were collected and aliquoted into 1 ml cryovials and banked for metagenomic sequencing at −80ºC to analyze differences in microbiota prior to FMT therapy.

### Phase 2: FMT administration

**Figure 1.**
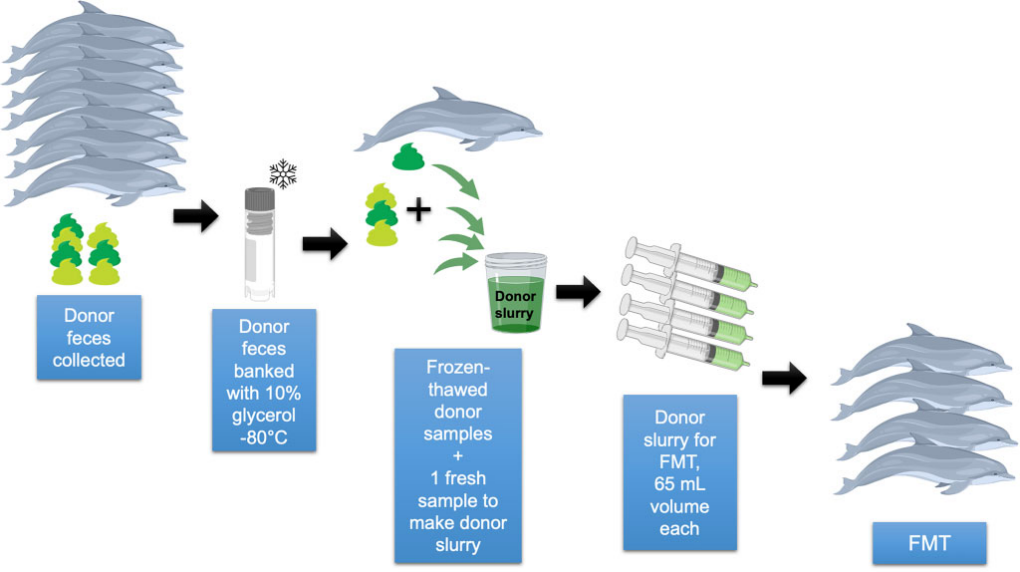
FMT preparation schematic. Feces were collected from multiple healthy, screened donor dolphins and added to the donor fecal bank. For FMT administration, multiple donor samples were combined with one fresh sample to achieve desired volume for FMT (65 ml). Donor slurries were then administered to recipient dolphins.

**Figure 2.**
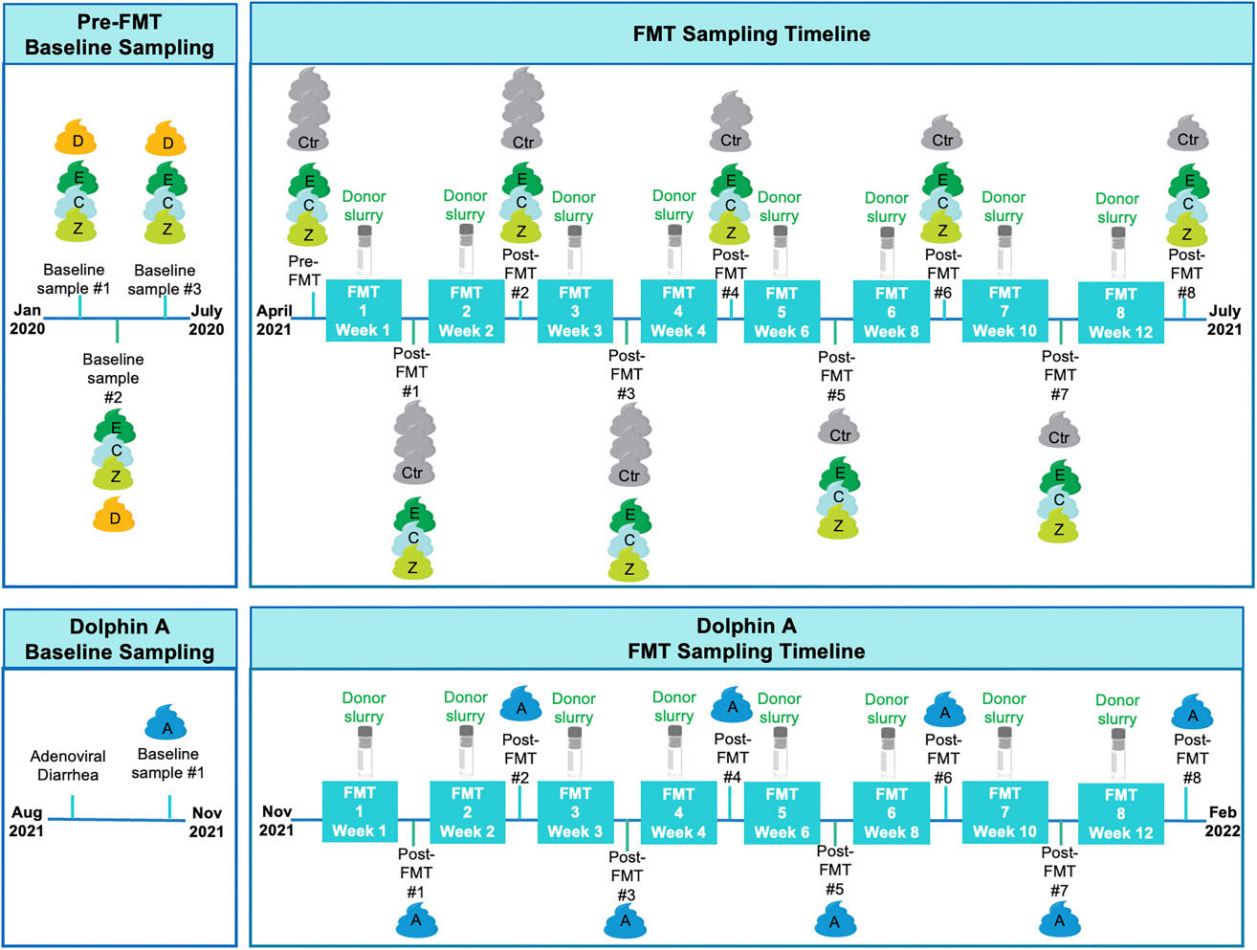
FMT administration and sampling timelines for Dolphins E, C, Z, and for Dolphin A. Dolphins E, C, and Z completed baseline samples in January–July 2020, and FMT administration in April–July 2021. Dolphin A completed baseline sampling in November of 2021 and received FMTs from the same donors in November 2021–February 2022. Abbreviations: FMT = fecal microbiota transplant, D = donor dolphins (*n* = 7), Ctr = control dolphins (*n* = 3), E = FMT recipient Dolphin E, C = FMT recipient Dolphin C, Z = FMT recipient Dolphin Z, and A = FMT recipient Dolphin A.

Eight total FMTs were performed on each recipient dolphin (Dolphins E, C, Z, and A): once weekly for 4 weeks, then every-other-week for four additional treatments. Dolphins E, C, and Z received the same treatment at the same times, while Dolphin A followed the same treatment regime but starting at a later date. FMTs were performed in the morning (07:30 a.m.–09:30 a.m.) with dolphins fasted overnight, in an effort to have the large intestine empty of fecal material. For each FMT, a “donor slurry” was created by thawing (warm water bath) banked donor fecal samples from multiple donors (three to six) and stirring to combine with fresh feces from at least one donor animal collected ∼1 h prior, to achieve a total 65 ml of donor slurry per recipient animal. Sterile saline was used as needed to achieve appropriate consistency for handling. An aliquot of the donor slurry was also banked for metagenomic sequencing with each FMT. Figures 1 and 2 illustrate the FMT protocol and sampling regime.

Donor slurries included feces from three to six distinct donors (of seven total donors). Several human and mouse studies have suggested that older donors have altered microbiomes compared to younger donors (Odamaki et al. 2016, Anand et al. 2017, Holmes et al. 2020) and may therefore be inferior FMT donors (D’Amato et al. 2020, Marotz et al. 2021), while a study in horses demonstrated no difference in young and geriatric donor horse microbiota (McKinney et al. 2020). Given that these data are lacking in dolphins, we chose to always mix the oldest donors’ (39 and 46 years) feces in the donor slurry with that of younger animals (7 to 20 years).

**Figure 3.**
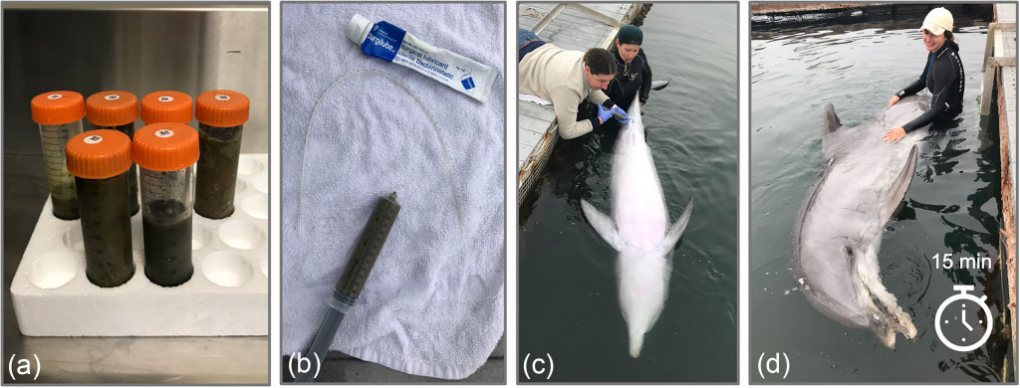
Dolphin FMT administration steps. (a) Frozen donor samples with glycerol thawing before being combined to create the FMT donor slurry. (b) Syringe of 65 ml donor fecal slurry, lubricant, and suction catheter for administration. (c–d) Maintaining lateral or ventral presentation for 15 min to increase contact time and prevent immediate defecation of FMT material.

To administer the donor slurry, under voluntary behavioral control, the recipient dolphin assumed a ventral presentation with the peduncle supported by a trainer and a 25-inch, 14Fr catheter was inserted into the rectum with sterile lubricant, ∼20 inches deep, in a manner similar to fecal collection (Fig. 3). The donor slurry was administered through the catheter using a 60 ml syringe, slowly over 3–5 min, followed by 5–10 ml of saline to flush remaining feces from the catheter. The trainers who held the dolphin in a lateral presentation or ventral presentation for 15 min in an effort to reduce immediate defecation and improve contact time and retention of the donor material within the colon; dolphins were fed during this 15-min incubation period.

### Post-FMT fecal sampling

A fecal sample was collected from each recipient dolphin in between the weekly (2–6 d post-FMT) and bi-weekly (6–13 d post-FMT) treatments and after the final FMT treatment (8–9 d post final FMT). Samples were collected from control dolphins on the same dates. Samples were aliquoted into a 1 ml cryovial and banked at −80ºC until being processed for metagenomic sequencing.

### Recipient monitoring

Recipient dolphins were monitored comprehensively throughout the FMT administration, including daily assessments of appetite, behavior, task-based learning, diet, fecal quality, and frequency. Routine bloodwork (complete blood count, serum chemistry, erythrocyte sedimentation rate, and fibrinogen) and clinical appearance of the recipients were also monitored by veterinarians.

### Metagenomic sequencing

The University of California Microbiome Core performed nucleic acid extractions using previously published protocols (D’Amato et al. 2020). Briefly, extractions and purifications were performed using the MagMAX Microbiome Ultra Nucleic Acid Isolation Kit (Thermo Fisher Scientific, USA) and automated on KingFisher Flex robots (Thermo Fisher Scientific, USA). Blank controls and mock communities (Zymo Research Corporation, USA) were carried through all downstream processing steps. Input DNA was quantified using a PicoGreen fluorescence assay (Thermo Fisher Scientific, USA) and metagenomic libraries were prepared with Illumina DNA Prep kits (Illumina Incorporated, USA) following the manufacturer’s instructions and automated on epMotion automated liquid handlers (Eppendorf, Germany). Sequencing was performed on the Illumina NovaSeq 6000 sequencing platform with paired-end 150 base pair cycles at the Institute for Genomic Medicine (IGM) at the University of California San Diego.

### Bioinformatic and statistical analysis

Raw FASTQ files of sequences were host-filtered against a reference dolphin genome (NCBI Reference Sequence: NC_047034.1) and aligned using the alignment tool Bowtie2 (Langmead and Salzberg 2012). Host-filtered files were uploaded and adapter-trimmed in the web-based bioinformatic platform Qiita (Gonzalez et al. 2018). Next, the files were put through the Woltka classification pipeline (Zhu et al. 2022), generating species-level taxonomic profiles of the samples using the Web of Life 2 (WoL2) reference database (Zhu et al. 2019).

Species-level taxonomic profiles were rarefied to a depth of 1070 operational genomic unit (OGU) reads to account for uneven sampling depth. Statistical analysis and visualization were performed on the resultant taxonomic and functional annotations using QIIME2 2023.2 (Bolyen et al. 2019) and R version 4.2.2 via the phyloseq package (McMurdie and phyloseq 2013). Alpha diversity values (within-sample diversity) were calculated using the Shannon’s diversity index. Analyses of beta diversity (between-sample diversity) were performed using unweighted UniFrac, weighted UniFrac, and/or Bray–Curtis community dissimilarity metrics (Lozupone and Knight 2005). Visualizations of ordinations were performed using principal coordinate analysis (PCoA), and tests for significance were performed using permutational multivariate analysis of variance (PERMANOVA).

## Results

FMT protocols for use in dolphins at the US Navy MMP were developed; a transrectal approach was chosen over oral administration due to the highly acidic dolphin stomach, with a protocol of weekly FMTs initially, which tapered to monthly over time. Due to the small volume of feces obtained from donor dolphins during collections, combinations of fresh donor feces and frozen feces with glycerol were utilized. These protocols were implemented in one pilot case, a young dolphin with dysbiosis as a sequela to viral enteritis, during August to December 2019 (Dolphin E). Throughout the therapeutic course of 12 FMTs, the dolphin was successfully weaned off of all medications, with complete resolution of inappetence, lethargy, and diarrhea. Following this successful pilot case, the current prospective study was undertaken.

Due to delays related to the global SARS-CoV-2 pandemic, there was a large gap in time between completion of baseline sample collection (January–July 2020) and performing FMTs for recipient Dolphins E, C, and Z (FMTs May–July 2021). Dolphin A developed dysbiosis later (August 2021) and had no delays in analysis or treatment (FMTs November 2021–February 2022). Figure 2 shows the sampling timeline schematic.

Dolphin A is the primary focus with regards to efficacy of FMT therapy given the timely clinical intervention. Given the confounding variables associated with delayed intervention for Dolphins E, C, and Z, their metagenomic data are reported, with an emphasis on the safety and limited interpretation with regards to efficacy.

Two of the three negative control dolphins were started on antibiotics during the study, one after the FMT #3 timepoint and the other after FMT #4 timepoint, at which point they were excluded from the study. Comparisons between negative controls and recipients were therefore limited, as there were fewer longitudinal samples from two of the three controls.

### Safety

Feces from the seven donor dolphins was deemed safe for FMT use based on both passing the screening protocol and metagenomic analysis showing no significant abundance of known or suspected pathogens.

All recipient dolphins tolerated FMT therapy well and no adverse effects were observed by veterinarians or trainers, including monitored bloodwork, ultrasound, appetite, behavior, task-based learning, fecal quality, and frequency. The three recipients who were not showing clinical signs at the start of the FMT process remained clinically normal, and the one dolphin with diarrhea and lethargy at the start of FMT showed full resolution of clinical signs throughout the treatment period.

### Microbial communities prior to FMT procedure

**Figure 4.**
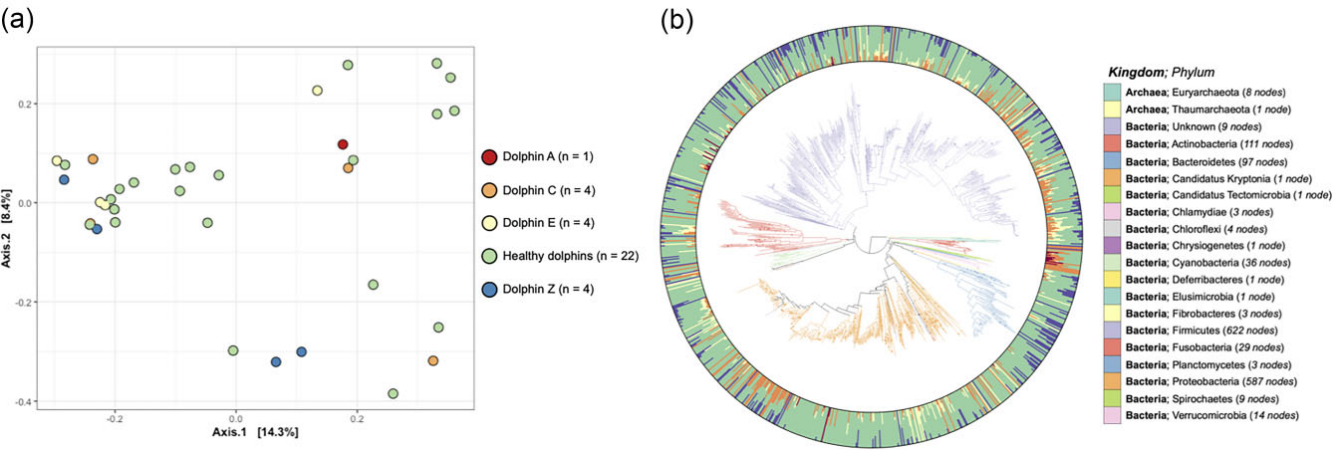
(a) PCoA plots of unweighted UniFrac distances describing the compositional variability of dolphin gut samples at baseline time points. (b) Phylogenetic tree of archaeal and bacterial species found in baseline samples, surrounded by a circular stacked bar plot describing the proportions of host dolphin groups that contain a given species. Node tips are color coded by phylum. Dolphin group code color is identical between the PCoA plot and the circular bar plot.

Characteristics of microbial communities in baseline samples were compared between recipients and healthy donors. Both recipient and donor microbiomes contained a diverse range of microbial taxa, including members of bacterial and archaeal kingdoms that encompassed at least 20 phyla (Fig. 4b). The communities were primarily dominated by the bacterial phyla *Firmicutes* and *Proteobacteria*, which typically composed >80% of sampled reads regardless of sample type. Other relatively abundant phyla included *Actinobacteria* and *Candidatus Kryptonia* (Supplementary Fig. S1). There were no significant differences in per-sample alpha diversity values between the two groups (*T*-test with Shannon*'*s diversity index, *T*-statistic = −0.360, *P*-value = .721), which measure both the number and evenness of taxa in each sample. Similarly, there were no significant differences in community composition centroids between the two groups (PERMANOVA with unweighted UniFrac, *R*^2^ = 0.02412, *P*-value = .275), nor in beta dispersion values within the groups (PERMDISP with Unweighted UniFrac, *F*-statistic = 0.0148, *P*-value = .1883). The bacterial community variability within donors and recipients was not significantly different than the variability between the groups, indicating that the microbial communities of donors and recipients were not significantly different at the start of the trial (Fig. 4a). Grouping baseline samples by dolphin host identity may better describe variability in community composition compared to the healthy/recipient grouping, although this was not significant either (PERMANOVA with unweighted UniFrac, *R*^2^ = 0.404, *P*-value = .052). Taken together, these results indicate that dolphin microbiomes were similarly variable regardless of donor/recipient status, and no consistent signature of either a healthy or a dysbiotic dolphin microbiome was observed.

### Microbial communities of donor FMT samples

**Figure 5.**
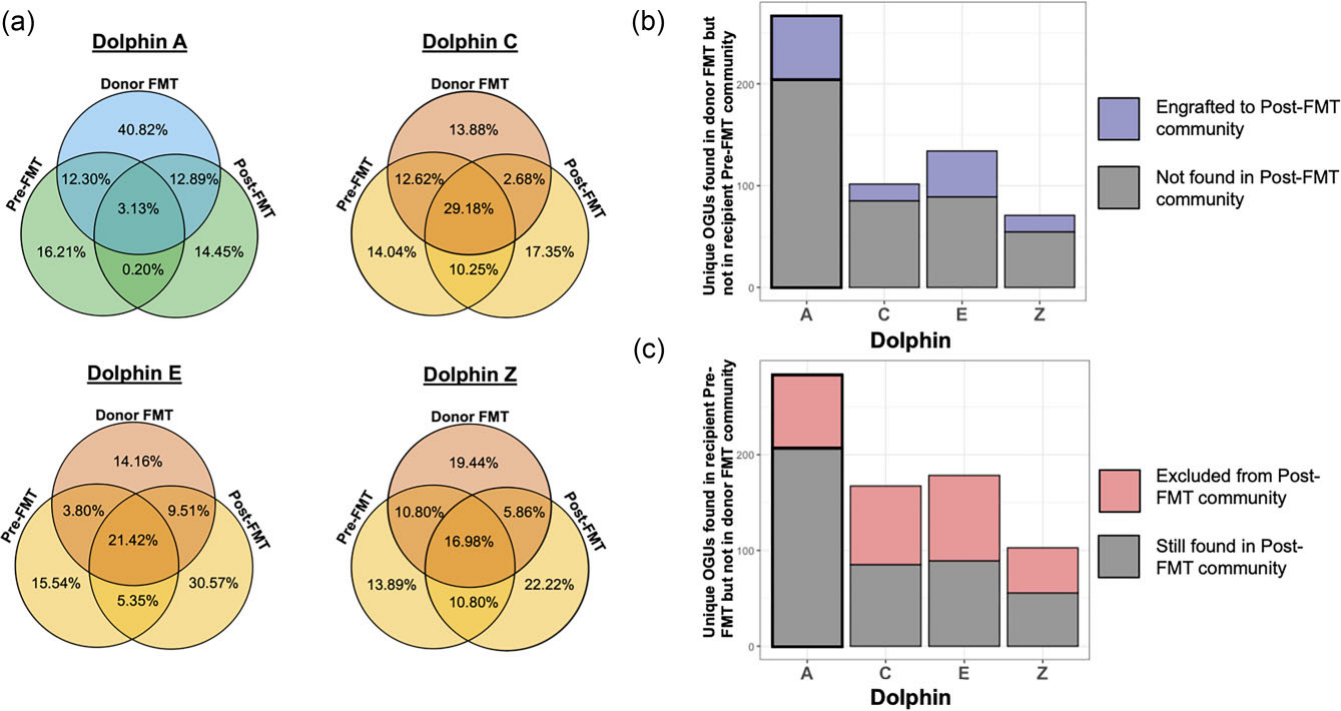
(a) Percent of unique microbial taxa that were found in either one or any combination of pre-FMT, donor FMT, or post-FMT sample, for each donor-recipient pairing. (b) Stacked bar plot of unique microbial taxa found in donor FMT communities but not in recipient pre-FMT communities, color coded by their presence post-FMT. (c) Stacked bar plot of unique microbial taxa found in recipient pre-FMT communities but not in donor FMT communities, color coded by their presence post-FMT.

**Figure 6.**
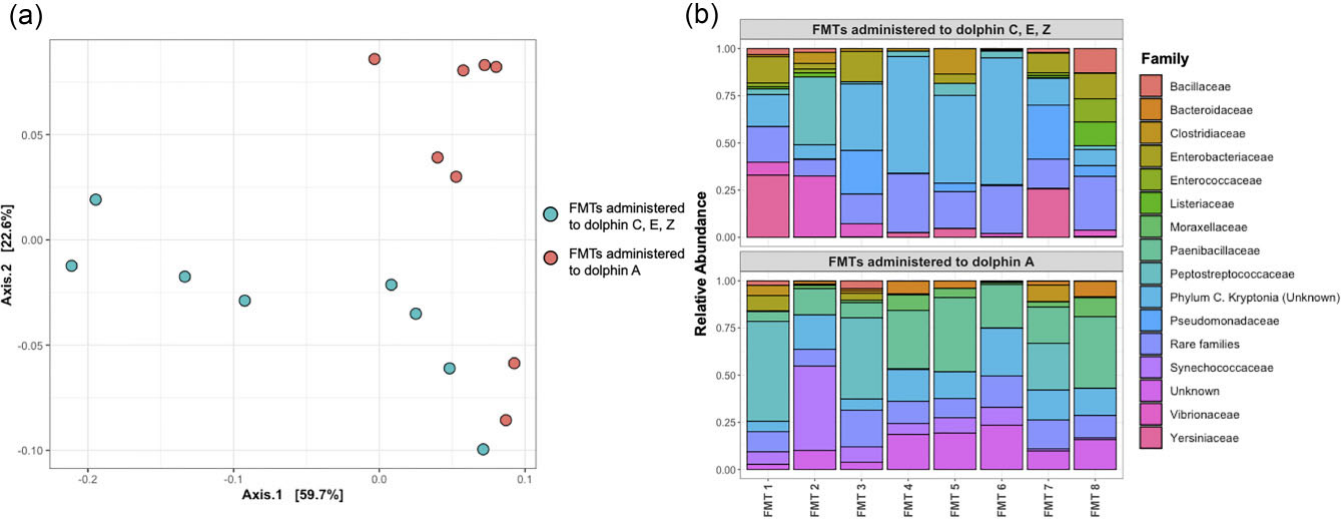
(a) PCoA plot of weighted UniFrac distances between FMT donor slurry samples, color coded by FMT group. (b) Stacked bar plots describing the taxonomic community composition of FMT donor slurry samples introduced to recipient dolphin gut communities, binned at the family level.

Microbial community characteristics of the FMT donor slurries were examined. Donor FMT communities contained a substantial number of microbial taxa that were not found in the initial baseline communities of the respective recipients (Fig. 5a). PERMANOVA tests used to determine whether there were significant differences between FMT communities administered to the first set of recipients (Dolphins C, E, and Z) and the second (Dolphin A) gave mixed results, depending on the community dissimilarity metric used. When using unweighted UniFrac distances, there were no significant differences in community group centroids as a function of recipient group (*R*^2 ^= 0.081, *P*-value = .139). However, there were significant and substantial differences when using other distance metrics, such as weighted UniFrac (*R*^2^ = 0.303, *P*-value < .001), and Bray–Curtis (*R*^2 ^= 0.266, *P*-value < .001) (Fig. 6a). This suggests that differences between microbial communities in the FMT groups are driven more by variation in relative abundance of species present (included in weighted UniFrac and Bray–Curtis metrics), rather than the variation in presence/absence of certain microorganisms. A potential confounding factor, which may have contributed to the lack of significant results with unweighted UniFrac distances is the notably high level of variability occurring within FMT samples of the same phase (Fig. 6b), which reduced the statistical power available given the limited sample size.

### Recipient microbial community changes following FMTs

The microbial community changes of recipient microbiomes were examined by comparing recipient fecal samples before and after the first FMT treatment to the FMT donor slurry microbiome. For this study, engraftment is defined as the number of OGUs (see Zhu et al. 2022) absent in the recipient pre-FMT community but present in the donor FMT community and subsequently, present in the recipient post-FMT community. Exclusion is defined as the number of OGUs present in the recipient pre-FMT community that are absent in the donor FMT community and subsequently, absent in the recipient post-FMT community (Fig. 5). The success rate of engraftment following FMT was relatively variable across recipients, with 17 to 62 newly introduced taxa engrafting, which translates to 20%–33% engraftment after the first FMT for each recipient dolphin. There were 62 instances of engraftment out of 205 introduced taxa (30%) in Dolphin A, 17 instances of engraftment out of 85 introduced taxa (20%) in Dolphin C, 45 instances of engraftment out of 134 introduced taxa (34%) in Dolphin E, and 17 instances of engraftment out of 55 introduced taxa (31%) in Dolphin Z (Fig. 5b). On the other hand, there were 79 instances of exclusion out of 285 excludable taxa (28%) in Dolphin A, 82 instances of exclusion out of 167 excludable taxa (49%) in Dolphin C, 89 instances of exclusion out of 178 excludable taxa (50%) in Dolphin E, and 47 instances of exclusion out of 103 excludable taxa (46%) in Dolphin Z. Dolphin A, the only dolphin with clinical symptoms at the time of the study, had the lowest % overlap between pre- and post-FMT samples, indicating the largest changes due to FMT therapy. Community dissimilarities between donor and recipient microbiota were assessed after each FMT for each dolphin using unweighted UniFrac distance, and no clear pattern or directionality was observed to indicate an additive effect of the FMTs (Supplementary Fig. S2).

**Figure 7.**
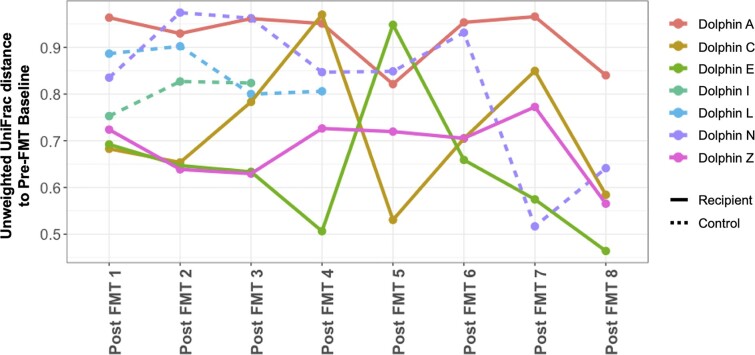
Time series plot of unweighted UniFrac distances examining community dissimilarity of post-FMT time points for each recipient and control dolphin, relative to its respective baseline community collected prior to FMT administration.

Notably, the amount of microbial community variation over time following the initiation of FMT treatment was comparable between those of FMT recipients (Dolphins A, C, E, Z) and those of the control dolphins who did not receive any treatment (Dolphins I, L, N), as measured by beta dispersion (*T*-test, *P*-value = .533). Complementarily, beta distance from baseline analyses examining unweighted UniFrac distances of post-FMT time points relative to each dolphin’s baseline community revealed similar community variation over time for both recipient dolphins and control dolphins (Fig. 7). Inference is limited due to the fact that two of the three controls were excluded from the study due to antibiotic use after the third and fourth time points.

### Tracking the microbiome dynamics of Dolphin A throughout the FMT process

**Figure 8.**
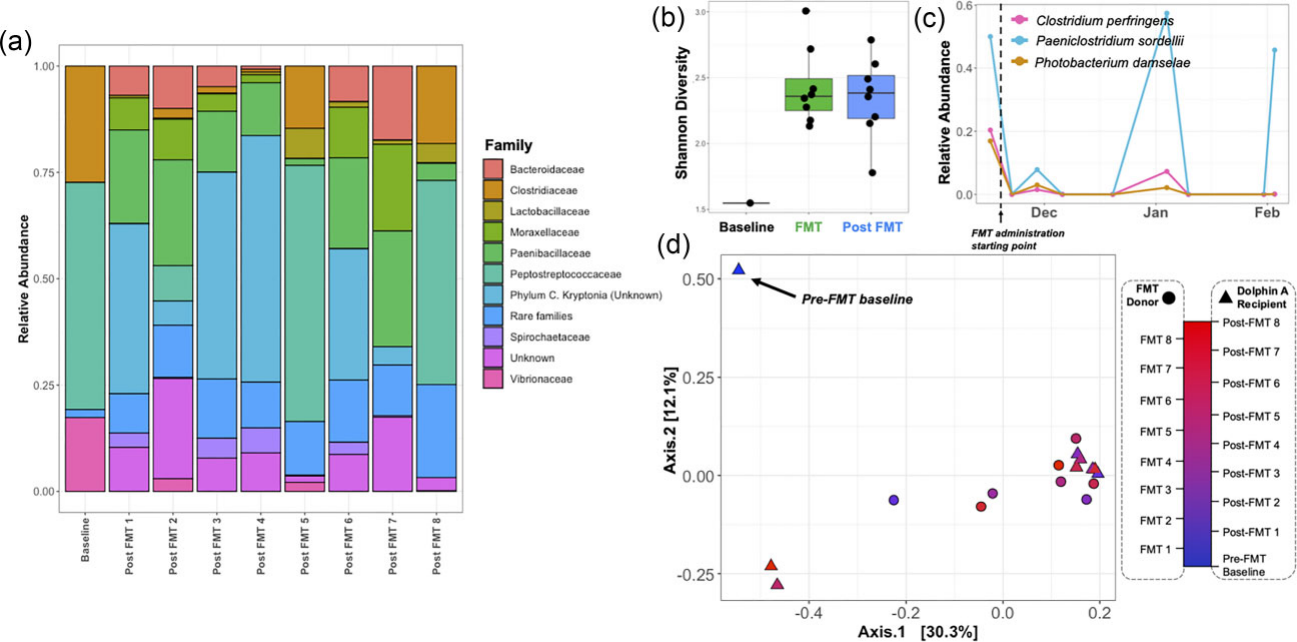
(a) Taxonomic bar plots describing the community composition of Dolphin A through time, binned at the family level. (b) Box plots comparing Shannon index values of pre-FMT, FMT, and post-FMT samples for Dolphin A. (c) Time series plots examining changes in the relative abundance of *C. perfringens, Pae. sordellii*, and *Ph. damselae* through time in Dolphin A’s samples throughout the study. (d) PCoA plot ordinating unweighted UniFrac distances of Dolphin A samples relative to FMT donor samples, color coded by time point. Black arrow indicates Dolphin A’s pre-FMT sample.

In this study, Dolphin A was the sole recipient with clinical signs of diarrhea and lethargy due to adenoviral enteritis prior to treatment and timely intervention with FMT therapy (no pandemic-related delays), and therefore, the microbial dynamics of his FMT therapy were investigated in closer detail. Dolphin A’s pre-FMT baseline alpha diversity was among the lowest of all baseline samples, with a Shannon index of 1.548 (Fig. 8a). Taxonomic annotation of the baseline sample revealed a community primarily dominated by three bacterial species (87.29% relative abundance total): *Paeniclostridium sordellii* (50.00% relative abundance), *C. perfringens* (20.37% relative abundance), and *Photobacterium damselae* (16.92% relative abundance) (Fig. 8a). Within the first 7 d following FMT procedure, Dolphin A experienced improvements in gastrointestinal symptoms, including improved appetite and energy, and medications were successfully tapered with no relapse in clinical signs. This outcome coincided with a sustained increase in alpha diversity to levels similar to what was found in FMT slurries (Fig. 8b), with the increase occurring immediately after the first FMT treatment. By FMT #5, Dolphin A was weaned off of all supportive medications (simethicone, maropitant citrate, ondansetron, and oral hydration), and diarrhea was no longer observed. Importantly, the relative abundances of the initially relatively abundant *Ph. damselae* and *C. perfringens* dramatically fell, with both species being undetectable in six out of eight post-FMT samples. The two species were detected in samples from post-FMT 2 and post-FMT 5 time points; however, relative abundances were much lower compared to the pre-FMT baseline sample (Fig. 8c). The third species that was initially dominant, *Pae. sordelli*, returned to initial levels at the same two time points, but with no resurgence of symptoms. These three species were either undetectable or only detectable at low abundances (<5% relative abundance) in the healthy donor sample communities. The overall composition of post-FMT samples in Dolphin A reflected a donor–recipient hybrid (Fig. 8d), and similarity to the donor community increased most following the first and fifth FMTs (Supplementary Fig. S2). It is important to note that this method cannot quantify absolute abundance, so we cannot identify whether increases in relative abundance of certain taxa reflect, e.g. an increase in their absolute abundance or a decrease in the relative abundances of other bacteria in the community.

## Discussion

This is the first peer-reviewed clinical study to examine the safety and efficacy of a fecal microbiota transplantation protocol in bottlenose dolphins (*T. truncatus*), to the authors’ knowledge. We developed and implemented a thorough donor screening protocol to ensure recipient safety. Results indicate that the protocol is effective and safe with no adverse effects observed in any recipient dolphins. Additionally, we described the microbial dynamics and clinical outcomes of FMT treatment for Dolphin A, who had acute adenoviral enteritis with diarrhea immediately prior to treatment. The data presented here demonstrate that FMT therapy can be done safely in dolphins with appropriate donor screening, and that FMTs can be effective in impacting the gut microbiota of recipients.

The described FMT donor screening protocol and administration protocol appeared safe. This study used a combination of fresh and frozen donor feces and rectal administration of FMT slurry, which was administered in eight repeated treatments over the course of 12 weeks. None of the recipient dolphins experienced negative clinical effects following FMT treatment, and are still doing well 24 months later, suggesting that the donor screening protocol was effective. As safety was the primary concern, the screening criteria and testing we employed for donor dolphins were equal to or perhaps more stringent than that utilized by most human stool banks (Krajicek et al. 2019). The results of in-house and reference laboratory tests performed on donor samples were affirmed with metagenomic analysis to ensure there was no significant abundance of pathogens in donor samples prior to FMT. The screening protocol of in-house and reference laboratory tests also proved to be thorough and acceptable for use, especially if metagenomic sequencing is not available or timely. No donor samples passed the screening protocol but failed metagenomic screening. Thus, donors can be effectively screened through the combination of clinical history fecal cytology, fecal float, culture, and PCR panels. However, with metagenomic sequencing becoming more widely available and turnaround times faster, this tool will become increasingly useful and will more accurately reflect the species present over standard culture methods. In-house cytology also proved to be valuable for a crude measure of bacterial diversity in the samples, and the cytological results mirrored the much more detailed results demonstrated with metagenomics of improved microbial diversity following FMT treatment. This was especially true for Dolphins E (pilot case) and A, who began with rare monomorphic cocci prior to treatment, and by the end of FMT treatments had a diverse population of bacteria, including cocci and rods of varying sizes.

In recipient dolphins, veterinarians monitored bloodwork, appetite, task-based learning, diet, fecal quality and frequency, full body ultrasound, and overall clinical appearance throughout the study. All recipient dolphins had normal bloodwork during the study period and no adverse changes were observed. Of note, when the pilot study dolphin, Dolphin E, received FMTs prior to this study, he initially had an inflammatory hemogram, which resolved throughout FMT treatment (without antimicrobials or anti-inflammatory medications).

The data showed that dolphin microbiomes can vary widely between individuals and over time.

Similarly to the gut microbiome dynamics characterized in humans (Lozupone et al. 2012, Lloyd-Price et al. 2016), the baseline microbial communities of the dolphins in our study had high interindividual variability, regardless of donor or recipient status. Moreover, each dolphin also displayed significant variability between collected time points, although this variability was generally lower than what was seen between individuals. We did not find any global compositional or diversity-related trends that clearly separated the microbial communities found in the donor group to those found in the recipient group. As such, this suggests the lack of specific microbial community members that need to be present or absent to cause a “healthy” or “unhealthy” microbiome state; rather, this state may be context-dependent on each individual host and on the set of interactions occurring within the microbial members within each community.

In the healthy donor and control dolphins in this study, microbial communities were dominated by the bacterial phyla *Firmicutes* and *Proteobacteria*, followed distantly by the phyla *Actinobateria* and *Candidatus Kryptonia*. This is consistent with the results of multiple previous studies using 16S rRNA sequencing approaches (Bik et al. 2016, Soverini et al. 2016, Suzuki et al. 2019, Robles-Malagamba et al. 2020), which also found *Firmicutes, Proteobacteria*, and/or *Actinobacteria* to be dominant bacterial phyla found in both dolphins in the wild and those under managed care, across several sampled regions around the world. However, the use of shotgun metagenomics over 16S in our study allowed for the additional identification of *Candidatus Kryptonia* as another common phylum of the dolphin microbiome. *Candidatus Kryptonia* is a recently discovered bacterial candidate phylum, which had eluded detection by 16S sequencing approaches due to rRNA primer biases, and is typically associated with geothermal spring environments (Eloe-Fadrosh et al. 2016).

Following FMT administration, recipient microbiomes were altered relative to pre-FMT baseline time points, typically increasing in community resemblance to donor samples. In all four recipient dolphins, we observed instances of species-level engraftment from the donor community to the recipient community after the first FMT. Such rates of engraftment, however, appeared to vary depending on the quantity of microbial overlap between pre-FMT recipient and FMT donor samples; the greater the difference in community composition between pre-FMT recipient and donor FMT communities, the greater the observed levels of engraftment in post-FMT recipient samples (Fig. 5).

FMT treatment in Dolphin A corresponded to clinical improvements of dysbiotic symptoms. Prior to FMT treatment, Dolphin A’s baseline community was dominated by three bacterial species: *Pae. sordellii* (50.00% relative abundance), *C. perfringens* (20.37% relative abundance), and *Ph. damselae* (16.92% relative abundance). All three bacterial species have been identified as potential pathogens in marine mammals and/or humans (Buck et al. 1987, Rivas et al. 2013, Kim et al. 2017). Immediately following the first FMT treatment, there was an increase in the alpha diversity of the recipient’s gut community and sharp reduction in the relative abundances of these three dominant species. This coincided with substantial clinical improvements in the dolphin’s dysbiotic symptoms, including improved appetite, decreased nausea, decreased diarrhea, and ability to discontinue supportive medications (antinausea, gas relief, and gastroprotectants). Sampling after subsequent FMT administrations demonstrated that *Ph. damselae* and *C. perfringens* remained undetectable or low in relative abundance throughout the treatment timeline, possibly suggesting a link between one or both these taxa and Dolphin A’s gut dysbiosis symptoms. Contrastingly, *Pae. sordellii* was found in high relative abundance (45.7%–57.3%) in two post-FMT time points after Dolphin A’s dysbiotic symptoms cleared, suggesting that in this instance, the relative abundance of this species may not have been linked to dysbiosis, as the symptoms did not reappear. At the time of this writing, over 2 years later, Dolphin A has not required further FMTs and is clinically normal.

We note that Dolphin A received intravenous antibiotics prior to FMT treatment due to severe neutropenia associated with viral enteritis. While the efficacy of antibiotic pretreatment (i.e. wiping out gut microbial communities prior to FMT treatment) is currently poorly understood with mixed results, in this instance, FMT treatment following antibiotic use was effective. Out of the recipient dolphins, Dolphin A experienced the greatest amounts of species-level engraftment and exclusion. As Dolphin A was the only recipient to receive antibiotics and also the only one with clinical symptoms at the time of the FMT, studies with larger sample sizes will be required to ascertain the relative importance of antibiotic pretreatment. Antibiotic use may also be responsible for the low alpha diversity values initially found in Dolphin A’s baseline community, prior to FMT. Studies have shown that pretreatment with antibiotics can improve FMT outcomes in humans with ulcerative colitis (Keshteli et al. 2017) and in mice (Ji et al. 2017), but other studies have suggested reduced or altered engraftment after antibiotic pretreatment (Freitag et al. 2019, Singh et al. 2022). Further work is needed to determine if antibiotic pretreatment is warranted in dolphins to eliminate pathogenic bacteria prior to initiating FMT therapy to restore intestinal flora.

Due to the aquatic nature of dolphins, we encountered several challenges, which helped to shape the dolphin FMT protocol that was employed. First, defecation can be very difficult to witness in dolphins living in sea pens, as normal dolphin feces are not solid like terrestrial mammals and quickly dissipates within the water. This makes it difficult for clinicians and trainers to know if a dolphin has recently defecated or if the dolphin is experiencing diarrhea unless it is witnessed at the right time or during a procedure or training session. Collecting fecal samples from healthy donor dolphins is therefore challenging, as their colons are oftentimes empty due to recent, unwitnessed defecation. When feces were successfully collected from donors, it was often a small volume. Inadequate volume of feces has been linked with poor outcomes in a systematic review of human FMT outcomes (Gough et al. 2011). In order to overcome this challenge, a donor fecal bank was created. Donor feces were combined with glycerol, which has been shown to improve survival of microbes during thawing (Satokari et al. 2015), and stored for future use. Small donor fecal samples were later combined to reach the desired volume (65 ml) for FMT use, termed the donor slurry. It is possible that multiple donor strategies are preferred to having a single donor in order to have a more diverse transplant product; in a recent meta-analysis of human FMT outcomes, multiple donor FMTs had better rates of success than single donor FMTs (Levast et al. 2023).

The FMT procedure was easy to perform in the water with trained dolphins. In the initial pilot FMT series with Dolphin E (prior to this study), he was not performing voluntary husbandry behaviors at the time and the first two FMTs were performed with the animal out of the water, which was also effective. By the third FMT, his behavior, appetite, and cooperation had improved so significantly that he performed the rest of the FMTs (4–12) in the water with voluntary participation. All of the FMTs performed during this study were performed in the water. In-water FMTs were preferred for ease on the dolphin and trainer, and due to potential for increased abdominal pressure when out of water potentially leading to expelling of FMT material sooner or to a greater extent than if neutrally buoyant in the water. It should be noted that even when performed in the water, dolphins often expelled a variable amount of FMT material during the 15-min incubation time following administration. This was noted during each FMT and the amount expelled was estimated to vary from 0% to 50% of the infused FMT volume. Interestingly, Dolphin A expelled ∼30% of the first FMT (i.e. he defecated an estimated 20 ml of the 65 ml instilled), yet he still showed a profound response to that first FMT treatment, resulting in a sharp increase in bacterial diversity that mirrored the donor microbial community. So, while it is disheartening to see a portion of the FMT material defecated out after administration, it would appear that as long as the entire volume is not defecated, it may still be efficacious. During Dolphin A’s FMT course, one lesson we learned was that if the dolphin remained in a fecal presentation at the water surface with the fecal catheter left in place in the rectum during the 15-min incubation time, 0% of the FMT material was expelled. This technique has since been employed in subsequent dolphin cases and seems to hold true for others, as well.

Additionally, the donor screening process was fairly time-consuming, and even in a large population of dolphins, very few donors successfully met the rigorous donor criteria. Given the concern for safety, we felt that thorough donor screening, to the same level or beyond that of human FMT standards was necessary to ensure positive outcomes. Dolphins were only considered as donors if they were not on any medications for 6 months prior, including ophthalmic antimicrobial drops and proton-pump inhibitors like omeprazole, as this been shown to alter gut flora (Imhann et al. 2016, Kostrzewska et al. 2017, Koo et al. 2019). Further, they must have had normal bloodwork, ultrasound, and no known illnesses within the last 6 months. Those who met the initial inclusion criteria then had their feces screened via fecal float, cultures, and PCR panels; heavy growths of *Clostridium spp*. or *E. coli* were then further tested for presence of their respective toxins. In humans, the few instances of death related to FMT therapy have been attributed to improper donor screening protocols, including an FDA Safety Alert in 2020, which reported adverse outcomes in patients administered toxigenic *E. coli* (US Food and Drug Administration 2020). A 2019 study also described transmission of an extended-spectrum beta-lactamase *E. coli* to two patients linked to a single FMT donor, which resulted in death of one person (DeFilipp et al. 2019). Therefore, it is imperative to utilize proper screening protocols before considering FMT, to ensure the risks are mitigated as much as possible. This thorough process is somewhat laborious and can be costly, but the importance is underscored. As metagenomics becomes more readily available, timely, and affordable, this will likely be beneficial to provide thorough and efficient screening tools, potentially from a single sample rather than multiple samples to multiple labs.

While the results of this study are encouraging, there were several limitations. First was the long delay between the initial baseline sampling and the administration of FMTs due to the global SARS-CoV-2 pandemic. Due to this delay, the three initial recipient dolphins were clinically normal by the time of the FMTs. The FMTs were still performed in these three young dolphins despite the lack of active clinical signs since they each had a history of early antibiotic use and chronic or intermittent diarrhea. Studies have demonstrated negative impacts of early antibiotic use on health and longevity in mice (Lynn et al. 2021), and FMT has been proven to alleviate early-life antibiotic-induced enteritis in piglets (Ma et al. 2022), so the FMTs in these three individuals were aimed at evaluating safety and potentially improving overall immune status for improved longevity. Dolphin A later became a recipient and was the only dolphin with clinical signs at the time of FMT treatment and a timely intervention (i.e. no pandemic-related delays). Given the small number of recipient dolphins, and unequal levels of clinical disease at the start of FMT, comparisons between these animals were limited. Statistical comparisons also were limited due to the small sample sizes of dolphins in each group. Additionally, two of the three control dolphins were withdrawn from the study prior to completion due to necessary antibiotic use (infections unrelated to the GI tract), which further hindered comparisons between control and treatment groups.

## Conclusion

In conclusion, this prospective clinical study describes a fecal microbiota transplant donor screening protocol and administration protocol in bottlenose dolphins under human care. The donor screening protocol proved to be safe in this study and no adverse effects were observed in four recipient dolphins. Recipients showed varying degrees of engraftment of introduced donor microbes following FMT, but generally demonstrated a recipient–donor hybrid microbiota. In one dolphin with diarrhea and lethargy due to viral enteritis and intravenous antibiotic use, the administration protocol and frequency described resulted in resolution of clinical signs and improvement in microbial diversity that lasted for 2 years after study conclusion. Further work is warranted with larger sample sizes to continue investigating the optimal protocol and efficacy of FMT in dolphins.

This was an imperfect clinical study and future studies with larger samples sizes are warranted to continue evaluating FMT efficacy in dolphins. The lessons learned and initial promising data described in this study are shared so that they may serve as building blocks for future research in marine mammal FMT therapy. Future studies in cetaceans could compare oral and rectal routes of administration, optimal length of FMT therapy or number of treatments, and could include longer longitudinal sampling after FMT therapy to better understand the variability of the dolphin microbiome over time. In human and lab animal medicine, FMT is emerging as a novel treatment for a variety of diseases outside of the GI tract, including depression, obesity, autism, diabetes mellitus, metabolic syndrome, Parkinson’s disease, multiple sclerosis, cirrhosis, and cancers, among others. Future studies in marine mammals could also explore FMT as a treatment option for nongastrointestinal diseases.

## Supplementary Material

lxae026_Supplemental_File

## Data Availability

The datasets generated and/or analyzed during the current study are available in the European Bioinformatics Institute repository, [PRJEB71372 ERP156176]. Additionally, sequencing data and processed tables and taxonomy assignments are available through QIITA under study ID 14869. The animal participation in this study is in line with the ARRIVE guidelines. Samples from animals were collected during their routine clinical care and under the authority codified in the US Code, Title 10, Section 7524. Secretary of Navy Instruction 3900.41H directs that Navy marine mammals be provided the highest quality of care. The MMP is accredited by AAALAC International and adheres to the national standards of the U.S. Public Health Service Policy on the Humane Care and Use of Laboratory Animals and the Animal Welfare Act.
