# Supplementary material for: Evaluation of the safety and efficacy of fecal microbiota transplantations in bottlenose dolphins (Tursiops truncatus) using metagenomic sequencing
Source: J Appl Microbiol. 2024 Feb 1;135(2):lxae026. doi: 10.1093/jambio/lxae026 (PMC10853691; doi:10.1093/jambio/lxae026)
Supplement: lxae026_Supplemental_File [file lxae026_supplemental_file.docx]

**Figure S1:**


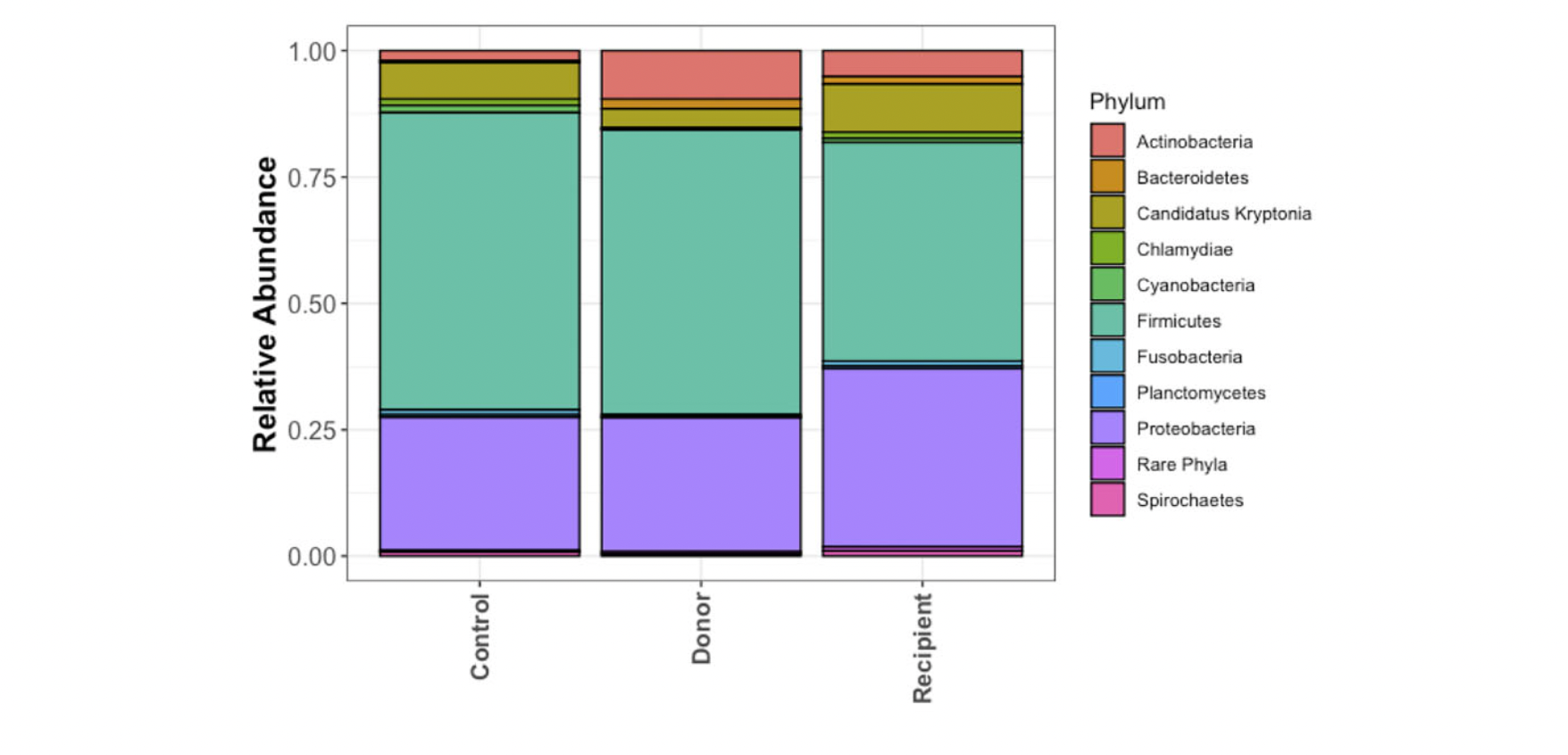


Supplementary Figure S1: Stacked bar plots describing the taxonomic community composition of donor baseline samples, recipient baseline samples, and control samples, binned at the phylum level.

**Figure S2:**


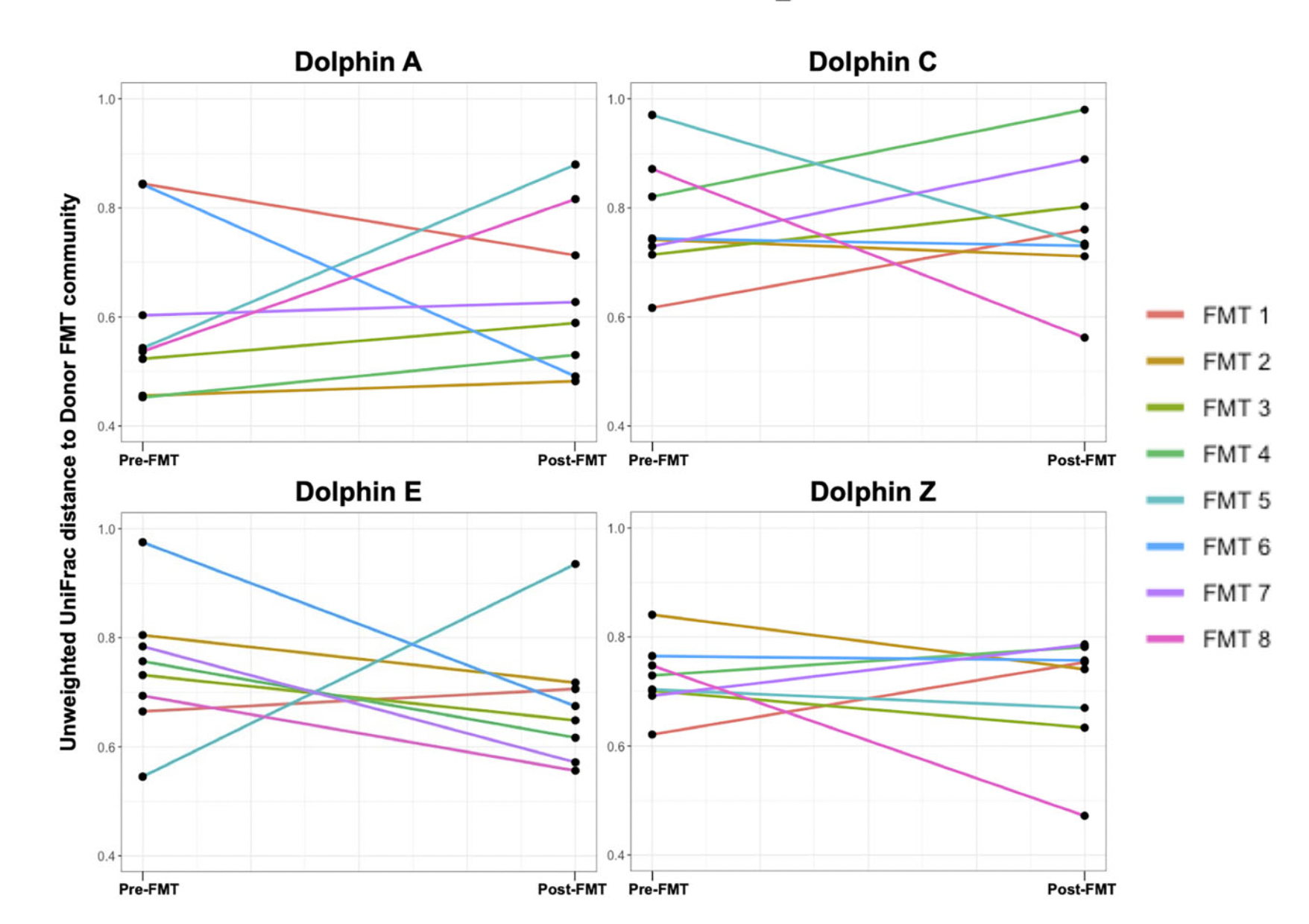


Supplementary Figure S2:  Plots examining Unweighted UniFrac distances to of pre-FMT and post-FMT recipient samples relative to the FMT donor sample, for all time points and for all recipient dolphins. Lines with negative slopes indicate increasing community similarity to donor FMT following treatment, and lines with positive slopes indicate decreasing community similarity to donor FMT following treatment.
